# Ultrasonic Extraction of Tropane Alkaloids from *Radix physochlainae* Using as Extractant an Ionic Liquid with Similar Structure

**DOI:** 10.3390/molecules24162897

**Published:** 2019-08-09

**Authors:** Alula Yohannes, Baohui Zhang, Bing Dong, Shun Yao

**Affiliations:** 1 School of Chemical Engineering, Sichuan University, Chengdu 610065, China; 2 School of Pharmacy, Hubei University of Chinese Medicine, Wuhan 430065, China

**Keywords:** *Radix physochlainae*, tropane alkaloids, tropine ionic liquids, ultrasonic extraction

## Abstract

In this research, tropane alkaloids in *Radix physochlainae* were extracted by tropine-type ionic liquid (IL) aqueous solutions under ultrasound assistance, and N-propyltropine hexafluorophosphate ([C_3_Tr][PF_6_]) was found to be the most ideal IL in this extraction mode after comprehensive screening. When 0.03 mol/L [C_3_Tr][PF_6_] aqueous solution was chosen as the extraction solvent, the solid-liquid ratio of raw material powders and ionic liquid aqueous solution was 1:20 (g/mL), ultrasonic power was 90 W and extraction time was 30 min, the extraction efficiency of tropane alkaloids has reached 121.3%. Compared with common heating extraction, it can further shorten the extraction time, improve extraction efficiency and decrease IL consumption. Furthermore, extraction mechanism together with potential toxicity of IL have been explored and discussed.

## 1. Introduction

Since the last decade, the extraction of natural products driven by new technologies is still a hot research topic, attracting more and more attention from academia and industry [1,2]. As a kind of neoteric solvent, the use of ionic liquids (ILs) has been frequently reported in this field for extraction objects that mainly include alkaloids, flavonoids, terpenoids, phenylpropanoids, polysaccharides, organic acids and others [3]. Compared with traditional organic solvents, ionic liquids can improve the extraction efficiency of bioactive constituents to a higher extent. They can form hydrogen bonds with the cellulose of plant material cells, strengthen mass transfer and interact with target molecules through noncovalent binding, so that more active components will be extracted efficiently from the internal cells. Besides that, the solubility of ILs can be improved by adjusting the combination of anions and cations to satisfy the interaction with different substances, thereby improving the separation efficiency. More importantly, they can also be recovered through back extraction, crystallization, adsorption or electrochemical methods after extraction. It should be acknowledged that ILs are becoming more and more popular in this area and the review of Ventura and colleagues has comprehensively summarized related basic trends in recent decades [4], which prove they have broad application prospects with obvious advantages over traditional solvents.

Recently, many alkaloids have been successfully extracted by various kinds of ionic liquids. Cao and coworkers [5] used imidazole ionic liquids based on ultrasound-assisted extraction technology to extract piperine from white pepper, and found that as the hydrophilicity of the ionic liquid increased, the extraction efficiency of piperine also increased up to 83.4%, higher than the 75% achieved using methanol. Besides that, a new method for the extraction of N-demethyllothine, O-demethyllothine and nuciferine by ionic liquid microwave-assisted extraction was developed [6]. It was found that [C_4_mim][Br] which is acidic and more conducive to the formation of salts with free alkaloids showed the most ideal extraction performance for these three alkaloids. In order to investigate the influence of cations and anions with different acidity on the extraction efficiency, eight kinds of 1-alkyl-3-methylimidazolium ILs with [HSO_4_]^−^, [H_2_PO_4_]^−^, [Br]^−^, [CH_3_SO_3_]^−^, [BF_4_]^−^, [PF_6_]^−^ or -SO_3_H substituted cation ([PSmim]^+^) have been synthesized by our group and applied to extract berberine under 100 W ultrasonic irradiation [7]. As a result, a solution of the acidic IL [PSmim][H_2_PO_4_] was found as the most efficient, followed by [PSmim][HSO_4_]. In addition, the length of the alkyl chain on the cation of the ionic liquid will also greatly affect its hydrophilicity/hydrophobicity, thus further affecting the alkaloid extraction efficiency. For example, Ma and coworkers [8] found that as the carbon chain length of the cations in imidazolium bromides increased from ethyl to octyl, the hydrophobicity of the cations increased gradually, and the extraction efficiency of camptothecin increased accordingly. This is because the hydrophobicity of lipid-soluble alkaloids creates a hydrophobic interaction with the ILs, the main driving force in the extraction process. Moreover, 5% [C_4_mim][Br] aqueous solution was proved to have same extraction efficiency for galantamine in comparison with H_2_SO_4_, but with a significant reduction in extraction time (from 15 h to 1 h) [9]. The appearance of more ILs and different successful methods is to be expected in this field, which is beneficial to analyze and summarize the extraction mechanism and interaction rules between ILs and target alkaloids.

In our previous study, it was found the tropine-type ILs have good selectivity in the extraction of tropane alkaloids, which that have the same structural nucleus with the extractant [10]. A generalized “like dissolves like” rule based on structural similarity has been preliminarily proved and satisfactory extraction efficiency was obtained in water-bath heating mode. Moreover, the IL also can be easily recovered at the end of extraction [11]. Besides that, it is necessary and valuable to explore the dissolution behaviors of targeted alkaloids in similar ILs with other efficient extraction modes. Furthermore, potential dissolution of other non-alkaloid constituents and toxicity of ILs also needs to be studied. In the following research, tropane alkaloids in *Radix physochlainae* were extracted by ionic liquid aqueous solutions under ultrasound assistance, and then various ILs were compared and the effects of the extraction conditions were investigated. Furthermore, as an important supplement to previous studies, all the findings were discussed to provide the necessary foundation for developing a complete methodological system and large-scale application of related ILs.

## 2. Results and Discussion

### 2.1. Screening of Ionic Liquids

**Figure 1:**
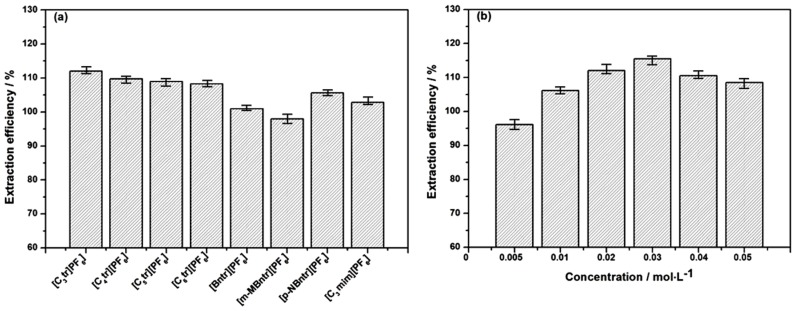
Extraction efficiency of various ILs (**a**) and concentrations (**b**).

In our previous investigation, [C_3_Tr][PF_6_] was found as the most ideal in common heating extraction (55 min in 75 °C water bath) [9]. Numerous results have shown that the structure of ionic liquids can affect their physical properties and dissolving capacity [12] thus resulting in different extraction performance for related constituents. In the following study, the extraction efficiency of total tropane alkaloids was compared by using seven kinds of tropine ionic liquids and one kind of imidazolium IL, which included [C_3~6_Tr][PF_6_], [C_3_mim][PF_6_], [Bntr][PF_6_], [*m*-MBntr][PF_6_] and [*p*-NBntr][PF_6_] (see Table 1). The hydrophobic anion of PF_6_^-^ has been proven to very suitable for the extraction of those low-polar constituents such as tropane alkaloids, so only the change of cation in ILs was considered in this section, which could provide different intermolecular interaction. The extraction results of these different ionic liquids are shown in Figure 1a. It can be seen from the graph that the extraction efficiency of [C_3_Tr][PF_6_] is still the best, which indicates different extraction modes have little influence on the overall performance of the extractants. The same solvent could obtain different results under varied conditions, but the order of different solvents will not change significantly under the same conditions. Therefore, [C_3_Tr][PF_6_] aqueous solution was selected as the most appropriate extractant for the following studies.

**Figure 2:**
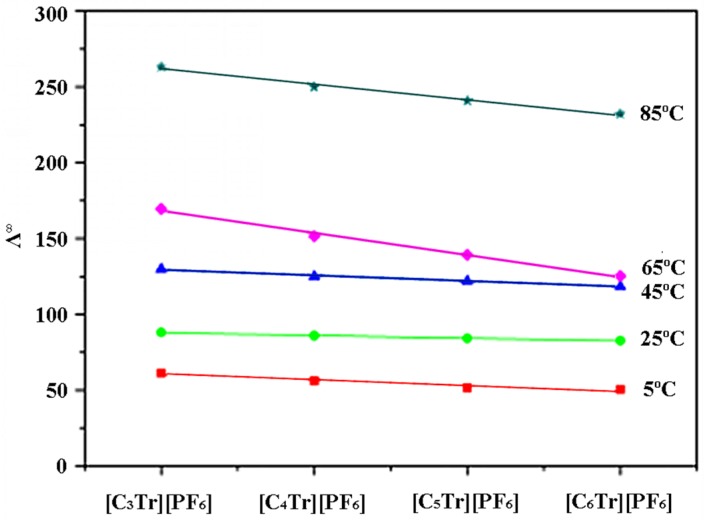
Linear fitting between limiting molar conductivity (Λ^∞^) and alkyl length on IL cations.

In order to explain the differences in extraction performance based on the essential features of the ionic liquids, four ionic liquids [C_3~6_Tr][PF_6_] with different alkyl chain length were selected to comprehensively measure their conductivity at different temperatures (Table 2). Combined with the data at room temperature reported in a previous study [12], it is not difficult to find that the order of conductivity is consistent with that of their extraction efficiency. With the increase of the alkyl chain length on the tropane cation nucleus, the limiting molar conductivity of ionic liquids will decrease (see Figure 2). This is because when the alkyl chain is longer, the volume of cations is greater; so their free movement rate in solution will decrease accordingly, resulting in the decrease of their limiting molar conductivity. Moreover, at the same temperature, the association constant of tropane ionic liquids is lower than that of imidazolium ionic liquids because of the lower surface charge density [13], which results in a weak association between anions and cations. The freer the ions are, the higher the conductivity is, and the more ideal the extraction performance can be achieved.

Besides the types of ionic liquid, the concentration also has a great influence on the extraction results of the target alkaloids. Too little ionic liquid will lead to incomplete extraction, as extraction efficiency is not high; on the other hand, excessive ionic liquid will cause waste. Meanwhile a higher viscosity is also not conducive to mass transfer. Therefore, the ionic liquid [C_3_Tr][PF_6_] with ideal extraction performance was selected as the study object and the effect of its concentration (from 0.005 mol/L to 0.05 mol/L) in aqueous solution on the extraction efficiency of tropane alkaloids was studied. The results are shown in Figure 1b. It can be found the extraction efficiency increases with the concentration in the lower concentration range (<0.03 mol/L). A necessary concentration of ionic liquid is the cardinal guarantee for comprehensive destruction of the internal/intermolecular hydrogen bonds of cellulose molecules together with effective dissolution of target components. When the concentration of ionic liquids is higher than 0.03 mol/L, the extraction efficiency begins to decrease; at this time the viscosity of extractant will become significantly higher with the increase of [C_3_Tr][PF_6_] concentration [14] and then raw material powders cannot be well dispersed in the ionic liquid aqueous solution. As a result the extraction efficiency of alkaloids decreases accordingly. Furthermore, compared with the optimal IL concentration of 0.05 mol/L in common heating extraction, 0.03 mol/L is enough to obtain a satisfactory extraction result in ultrasonic mode because of the enhancement of mass transfer. In summary, the extraction assisted by ultrasonic wave will not change the extraction superiority of [C_3_Tr][PF_6_] over other ILs, meanwhile it can reduce its consumption in actual application.

### 2.2. Effects of Extraction Conditions

**Figure 3:**
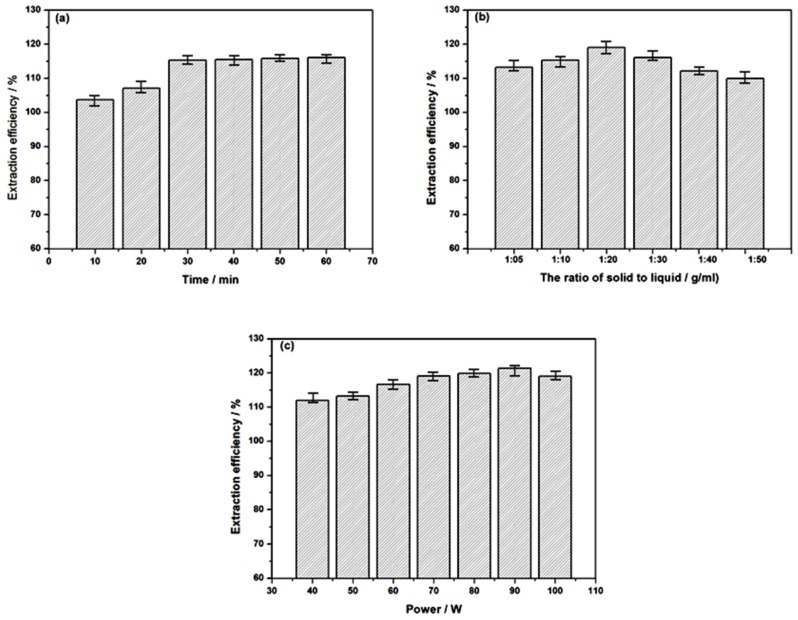
Effects of time (**a**), ratio of solid/liquid (**b**) and ultrasonic power (**c**) on extraction efficiency.

The extraction efficiency of tropane alkaloids with ionic liquid aqueous solution is directly affected by the extraction process and related conditions. In order to explore their influential roles and improve the extraction efficiency, the involved main factors were screened and investigated successively, which included extraction time, solid-liquid ratio and extraction temperature. In this section, the effect of extraction time was explored by the single factor method keeping the extraction temperature and other factors unchanged, and the relationship of different extraction times (10, 20, 30, 40, 50, 60 min) and the content of tropane alkaloids in the extract was studied. As shown in Figure 3a, the results prove that the ultrasonic extraction efficiency increases within the time range from 0 to 30 min, and then remains almost unchanged after more than 30 min. On the one hand, with the increase of extraction time, more and more soluble components of *Radix physochlainae* are dissolved and related extraction efficiency increases; on the other hand, when the tropane alkaloids have been fully dissolved, their content in the solution will no longer increase with the extraction time. Compared with the extraction time of 75 min in water-bath heating mode, 30 min is much shorter and more than half of the time can be saved.

The solid-liquid ratio is an important factor affecting extraction efficiency in solvent extraction processes. If mass transfer can be effectively strengthened, the denominator of solid-liquid ratio will become smaller and more extractant can be saved. In order to prove this trend, the extraction efficiency of tropane alkaloids was investigated under different solid-liquid ratio values (1:5, 1:10, 1:20, 1:3, 1:40, 1:50 g/mL). As shown in Figure 3b, the extraction efficiency increases first and then decreases with the increase of solid-liquid ratio. This is because the concentration difference between intracellular and external cells increases when the solid-liquid ratio becomes higher. The greater the driving force of mass transfer, the greater the internal diffusion speed, and more favorable the dissolution of alkaloids. Therefore, with the further increase of solid-liquid ratio, the extraction efficiency of alkaloids begins to stop rising. Because there is too much extractant, the energy of ultrasonic waves is absorbed by a large amount of solvent molecules as energy transfer medium in the extraction process and cannot completely act on the herbal material. At the same time, excessive solvent is not conducive to subsequent treatment after extraction, and 1:20 g/mL is enough for the following study as solid-liquid ratio, which is less than 1:35 g/mL used in common heating mode.

Ultrasonic irradiation can produce strong cavitation effects, mechanical vibration, perturbation effects, high acceleration, emulsification, diffusion, smashing and stirring effects, as a result the motion frequency and speed of molecules increase and the solvent penetration is strengthened. The entry of target components into the solvent can be accelerated, which promotes continuous dissolution of intracellular alkaloid constituents into [C_3_Tr][PF_6_] solution together with the whole extraction process. In this section, the effect of different ultrasonic power levels (40, 50, 60, 70, 80, 90, 100 W) on the alkaloid content in the extract is studied. As shown in Figure 3c, with the increase of ultrasonic power, the ionic liquids can be completely dispersed into the sample to promote the continuous dissolution of tropane alkaloids, but also increase the contact area between the herbal material and the solution, so the extraction efficiency increases; when the ultrasonic power exceeds 90 W, the alkaloid content in the extract begins to decrease, which should be ascribed to the thermal effect and the unstable ether/ester bonds in the structures of the target alkaloids beginning to break. The disadvantage of too high a temperature has also been found in common heating extraction, so the control of temperature should be considered for this kind of unstable constituents.

Through the above single factor experiments, when 0.03 mol/L [C_3_Tr][PF_6_] aqueous solution was chosen as the extraction solvent, the solid-liquid ratio of raw material powders and ionic liquid aqueous solution was 1:20 (g/mL), ultrasonic power was 90 W, and extraction time was 30 min, the extraction efficiency of tropane alkaloids from *Radix physochlainae* reached 121.3%, while the alkaloid content in the extract with the authoritative method in 2015 edition Chinese Pharmacopoeia (Ch.P.) was treated as 100%.

### 2.3. Association Effect between Extractant and Solutes

In order to further study the extraction mechanism and reveal the interactions between the ionic liquid and extracted alkaloids with same structural nucleus, here the association constant between them are calculated according to the equation:

$$\frac{1}{\Lambda_{m}}=\frac{1}{{\Lambda_{m}}^{\infty }}+{\frac{c\Lambda_{m}K_{a}}{{\left({\Lambda_{m}}^{\infty }\right)}^{2}}}_{}$$

where Λ*_m_* is the molar conductivity of [C_3_Tr][PF_6_] in prepared alkaloid aqueous solution with different concentrations (S·cm^2^·mol^−^^1^); Λ*_m_*^∞^ is the limiting molar conductivity of the IL in aqueous solution (S·cm^2^·mol^−^^1^); *c* is the volume molar concentration of ionic liquids in aqueous solution (mol·L^−1^); *K_a_* is the ion association constant (L mol^−1^). From this equation, the molar conductivity and the concentration of the IL are known in the experiments. If the curve is plotted in abscissa coordinate with 1/Λ*_m_* as the ordinate coordinate, the curve can be extrapolated and the association constant can be obtained through the slope of the straight line. The limiting molar conductivity and association constants of ionic liquids in alkaloid aqueous solutions at different temperatures are listed in Table 3.

As a result, when the experimental temperature is constant, the limiting molar conductivity of ionic liquids increases first and then decreases with the increase of alkaloid concentration. This is because the solvation of solution increases with the slow increase of alkaloid concentration in solution at the first stage, [C_3_Tr][PF_6_] dissociates into more free anions and cations, and their limiting molar conductivity increases; however, when the alkaloid concentration exceeds a certain value (inflection point), the limiting molar conductivity of ionic liquids decreases. This is because, on the one hand, the solution viscosity increases with the increase of alkaloid concentration, so the ion movement will slow down and the migration rate becomes lower, thus the limiting molar conductivity decreases; on the other hand, when the alkaloid concentration is increased in solution, it means that more alkaloid molecules interact with [C_3_Tr][PF_6_]. The molar conductivity of the IL decreases because the association between them results in the substances with poor conductivity. When the concentration of target alkaloids is constant, the limiting molar conductivity of the IL will increase with the increase of temperature. On the one hand, higher temperature accelerates the ion migration rate; on the other hand, higher temperature can weaken the electrostatic force between anions and cations of [C_3_Tr][PF_6_], and further destroys the hydrogen bond and van der Waals force between the ionic liquid and solvent. It can be said that raised temperature affect the interactions between ions or ions and solvent, and makes more free ions dissociated. Therefore, the limiting molar conductivity of the ionic liquid is positively correlated with temperature change.

### 2.4. Comparison with Traditional Extractant in Ultrasound Assisted Mode

**Figure 4:**
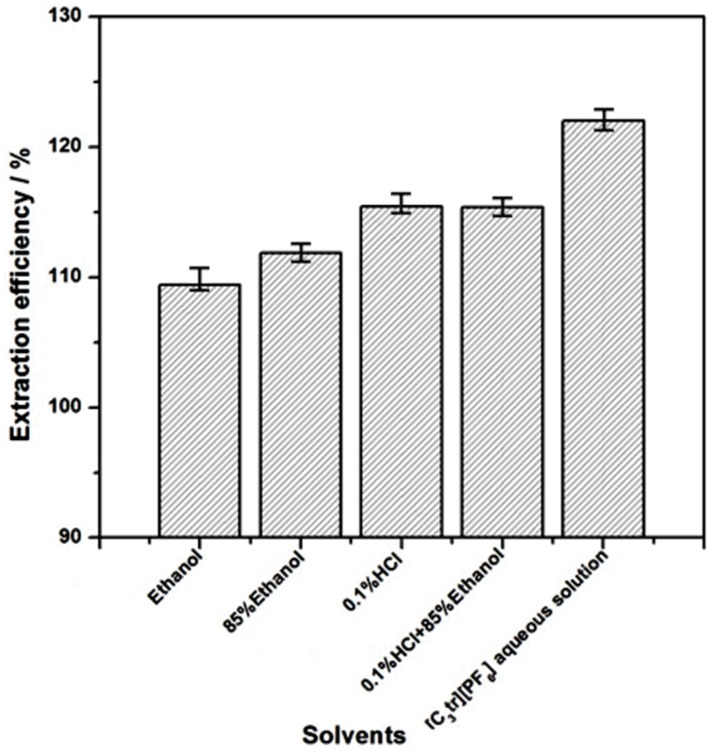
Comparison on extraction efficiency obtained by different solvents.

In order to validate the effectiveness of extracting alkaloids with ionic liquid aqueous solution under ultrasonic extraction mode and its advantage over traditional extractants, 0.1% hydrochloric acid + water, 85% ethanol + water, 85% ethanol + 0.1% hydrochloric acid + water ternary system were selected as reference solvents to extract tropane alkaloids from *Radix physochlainae*. Comparative experiments were carried out under the same ultrasonic extraction condition (solid-liquid ratio: 1:20 g/mL, ultrasound power: 90 W, extraction time: 30 min). Figure 4 shows that the extraction efficiency of target alkaloids by 0.03 mol/L [C_3_Tr][PF_6_] aqueous solution is obviously higher than that achieved by traditional solvents. That is, the ionic liquid aqueous solution is very suitable to extract these alkaloids with same structural nucleus, and its performance is better than that of traditional solvent systems. Among the latter, the extraction efficiency of acidic system is higher than that of neutral systems because the objects are alkaline, which basically accords with the trend found in common heating mode; however, [C_3_Tr][PF_6_] aqueous solution is superior to acidic systems, which indicates it has a special extraction mechanism and the acid-base interaction no longer play key role. In detail, it can provide more intermolecular interactions (electrostatic, H-bond and Van der Waals force) with target compounds and exhibit strong dissolving capacity; ideal conductivity and proper viscosity are very conducive to molecular diffusion. In addition, it can effectively interact with cellulose skeleton, which constitutes an important supporting structure for herbal raw materials, and then make it swell and porous. The mass transfer inside and outside cells is significantly enhanced. Previously, “Like dissolves like” theory referred to the overall solvation capacity of a solvent depends primarily on its polarity. Now the similarity of structure determines the selectivity of interaction. Moreover, compared with the traditional solvents, though the cost of ionic liquid is a little higher, its consumption during extraction is very low. Besides that, this kind of environmentally friendly solvent can also be recovered efficiently.

### 2.5. Dissolution of Non-Alkaloid Constituents under Ultrasonic Extraction Conditions

The simultaneous extraction of coexisting compounds could occur with ultrasound-based extraction due to the enhancement of mass transfer compared with the mild extraction method. According to previous study [15], the main bioactive compounds in *Radix physochlainae* are tropane alkaloids; besides them, scopoletin (7-hydroxyl-6-methoxycoumarin) is another kind of typical constituent, representative of natural coumarins. In common heating mode (≤75 °C), there was little scopolamine detected in the extract, and total alkaloids with satisfactory purity could be obtained. In order to explore potential change of its dissolving behavior in the extractant under ultrasonic conditions, a quantitation method for scopoletin was first developed according to [16] as follows: 10 mg of scopoletin standard was weighed and dissolved in a 100 mL volumetric flask with 2.5% sodium carbonate solution, and then 0.25, 0.3, 0.4, 0.45, 0.5, 0.52 mL of this solution was drawn into 10 mL volumetric flasks and diluted to the mark with 2.5% sodium carbonate solution, respectively. The absorbance was measured with UV spectroscopy at 343 nm as λ_max_ within 40 min, which was determined by full wavelength scanning (200~400 nm). The standard curve of scopoletin was developed on the basis of above solution concentration (x) and corresponding absorbance (y), which was determined as y = 30.382x + 0.4523 (R^2^ = 0.9991), and the linear range was 0.025~0.05 mg/mL. Secondly, the dissolution behavior of scopoletin in *Radix physochlainae* was investigated in 80% ethanol, 85% ethanol and 0.03 mol/L [C_3_Tr][PF_6_] aqueous solutions in ultrasound-assisted mode. In order to eliminate the interference of other factors, all of solid-liquid ratios of the three systems were 1:20 g/mL and the ultrasonic power was 90 W. The powders of raw material were fully mixed and extracted with the above systems under ultrasonic extraction for 30 min. After extraction and filtering, the pH value of 20 mL filtrate was decreased by 0.1% HCl aqueous solution in the range of 3~7 and then was further extracted by chloroform three times, while tropane alkaloids were ionized and remained in the extractive solution. After removal of chloroform under vacuum, the spectrophotometric method was used to determine the content of scopoletin in the extract at 343 nm. The results in Table 4 show that scopoletin can be extracted by the IL aqueous solution under the above ultrasonic conditions while its content in the extract (0.03 mol/L [C_3_Tr][PF_6_]) was still lower than that of 80% and 85% ethanol, and this is also aa advantage of IL over traditional solvents.

### 2.6. Characterization of Plant Residue after Ultrasonic Extraction and Extract Solution

**Figure 5:**
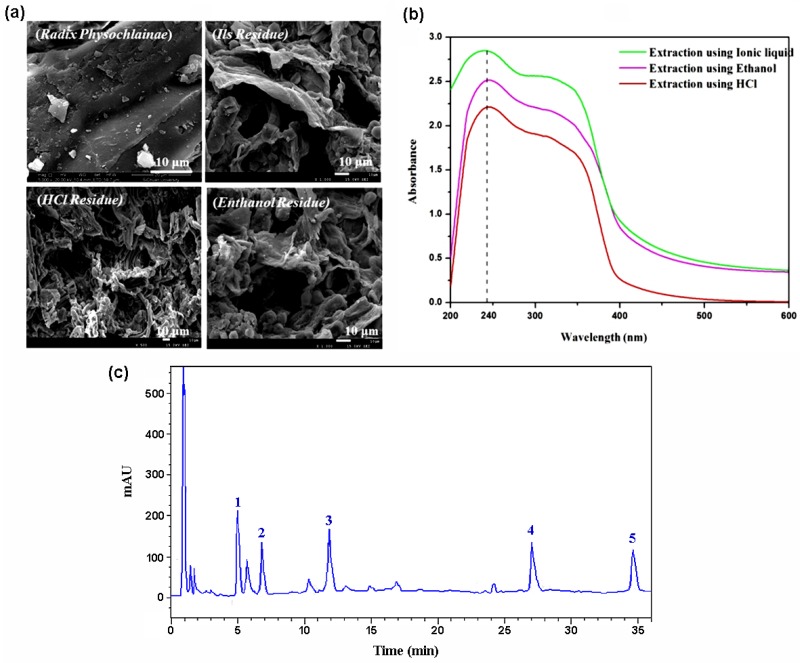
Comparison on SEM photos of herbal residue before and after extraction by different solvents (**a**), UV spectra for different extracts by various solvents (**b**) and chromatogram for five main alkaloids after extraction by IL (**c**, 1: anisodamine, 2: atropine, 3: scopolamine, 4: aposcopolamine, 5: scopoline).

The results of scanning electron microscopy for the observation of the residue extracted with three solvents in ultrasonic wave are shown in Figure 5a. Obviously, the surface morphology of the samples before and after extraction has changed distinctly, and the change is more significant than that under common heating extraction conditions. Before extraction, the structure of *Radix physochlainae* was relatively compact, and its surface was smooth and flat. After extraction with the three solvents, the cell walls were greatly damaged; the structure of herbal material became loose and many hollows and pores appear on the surface. Compared with the morphology of various residues after extraction by the three solvents, the surface of the sample extracted by ionic liquids has more holes than those extracted by ethanol and hydrochloric acid. Besides simple external destruction, the swelling effect of IL is more obvious than with the other two extractants, which is also very beneficial for substance exchange between inside and outside the cell membrane [17]. Under the influence of multiple actions, it shows that the structure of the herbal materials can be transformed to a greater extent by ultrasonic extraction under the above conditions, and IL molecules can enter into the plant cells and interact with the target alkaloids more easily. Furthermore, the ultraviolet spectra of the extracts obtained from three solvents are shown in Figure 5b. 230~270 nm is the characteristic π-π* absorbance band produced by the electronic transition of benzene ring in target alkaloids. It can be found the absorbance intensity of ionic liquid extract > the ethanol extract > the hydrochloric acid extract. Therefore, under the same extraction conditions, the extraction performance of ionic liquid [C_3_Tr][PF_6_] is the best. On the other hand, compared with the ultraviolet spectra of ethanol and hydrochloric acid extracts, the extract of [C_3_Tr][PF_6_] do not result in the increase or decrease of the absorption peaks, and the wavelength corresponding to the maximum absorption peaks do not show any red-shift and blue-shift. Peaks shape also shows no obvious change. In order to further prove the extraction results reflected by UV spectra, HPLC was used to analyze the chemical composition of the extract. As shown in Figure 5c, all the five main alkaloids appear in the chromatogram after ultrasound-assisted extraction by the IL. In summary, the employed ionic liquid as extractant cannot affect the structure of target alkaloids and extraction condition is very mild.

**Figure 6:**
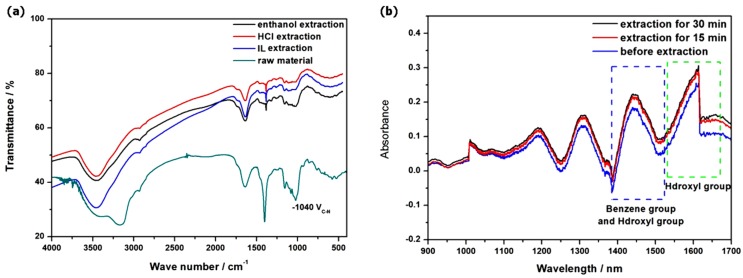
Comparison on IR spectra (KBr disc) of herbal residue before and after extraction by different solvents (**a**) and NIR detection on different time points during IL extraction (**b**).

Furthermore, FT-IR spectroscopy was used to characterize the dried powders of *Radix physochlainae* before and after ultrasonic extraction, which can indicate the chemical environment together with existence status of key groups in the structures of tested samples. The main chemical components of herbal materials are cellulose, hemicelluloses, lignin and active ingredients, in which cellulose is the main component. The infrared spectra of raw material before and after extraction are shown in Figure 6a, which indicates the absorbance intensity of bending vibration of -CH_3_/CH_2_ (1400~1425 cm^−^^1^) is higher than that of common cellulose; more importantly, the characteristic absorbance of stretching vibration of tertiary amine (ν_C-N_) in the structure of active alkaloids exists in the range of 1250~1020 cm^−^^1^. Compared with the raw materials, there re no new absorption peaks appearing in the IR spectra of the residue extracted with the three solvents, indicating that no chemical reactions occur during extraction. This can prove that ultrasonic-assisted extraction will not result in any significant modification of the skeleton/structure of raw materials together with target molecules. After extraction, the absorbance intensity of the characteristic functional group (tertiary amines of tropane alkaloids) decreases at the respective wavelength position, indicating that these solvents can effectively extract tropane alkaloids from *Radix physochlainae*. Comparing the three solvents, including ionic liquid, hydrochloric acid and ethanol, it is obvious that the intensity of characteristic ν_C-N_ signals is weakened after extraction with [C_3_Tr][PF_6_] and the extent of the decline is higher than that of the sample extraction with the other two extractants, indicating that the extraction result of tropane alkaloids from herbal material with the ionic liquid was ideal under the same extraction conditions from the other side. Furthermore, compared with the infrared spectra of the residue extracted in various modes, it is found that the difference of intensity change of the characteristic absorption peak (ν_C-N_) is less obvious in ultrasonic extraction than that in common heating extraction, which indicates that the extraction solvent has a greater influence on the extraction result in the latter mode. This result accords with the actual situation in many studies, that is when mass transfer is effectively strengthened, the difference between different solvents decreases.

Finally, in order to investigate the content variance of alkaloids in the extraction process after different durations, the absorbance of the whole extraction system in the range from 900 to 1700 nm was detected by near infrared (NIR) spectroscopy, which can be used in process analysis of the target constituents to study their dissolution dynamics. The results are shown in Figure 5b and it can be found in the near infrared spectra that the second-order double-frequency absorbance band of C-H is at 1150~1250 nm, the characteristic absorbance of benzene rings (1446 nm) and the first-order double-frequency doubling absorbance of hydroxyl groups (1430 nm) appear in the range of 1400~1500 nm; the hydroxyl group absorbance peak is near 1600 nm. The blue line, red line and black line represent the near infrared spectra after ultrasonic extraction for 0 min, 15 min and 30 min, respectively. Obviously, the near-infrared absorption intensity of the above groups increased with the prolongation of extraction time. Because all the structures of major tropane alkaloids in *Radix physochlainae* contain these groups, it proves that more and more target constituents are diffusing from the raw materials into the solution as the extraction proceeds. This method can be used to analyze the ultrasonic extraction progress by using ionic liquid [C_3_Tr][PF_6_]. Compared with the near-infrared spectroscopy of the extraction process in various modes, it is found that the absorbance increase of liquid system is more obvious in ultrasonic extraction than that in common heating extraction. This is because ultrasonic assisted mode is beneficial to extract more alkaloids within the same duration than heating mode.

### 2.7. Optical Rotation Detection for Potential Racemization

Most of tropane alkaloids have chiral centers and optical activity, and solvents and extraction conditions may affect their absolute configurations. For example, it was previously reported that they could be racemized under alkaline conditions. Scopolamine is an L-isomer; and during the extraction process, its majority will become atropine (its racemate). This is a potential problem that should be avoided in establishing new extraction processes. Here four samples are measured and compared to explore the potential effect of extractant on target alkaloids, and related automatic optical data are shown in Table 5. It can be found that under the same experimental conditions (mixing for 30 min in 90 W ultrasonic wave), the optical rotation values of the two systems of anisodamine (main compound in total alkaloids) + ionic liquid ([C_3_Tr][PF_6_]) and anisodamine + water are equal; the optical rotation value of the ionic liquid aqueous solution is 0, while the optical rotation value of the anisodamine + ammonia aqueous solution is decreased. This indicates that the aqueous solution of ionic liquid contains no optically active substances, and it is found that the uses of ionic liquid in the existence of anisodamine will not racemize the latter. In contrast, alkaline conditions can accelerate the racemization of anisodamine, which is consistent with the reported results, while ionic liquids can protect the chiral center of anisodamine and maintain its absolute configuration through intermolecular interactions. By comparing the optical rotation values of the four samples at different temperatures, it can be seen decreasing with the increase of temperature, indicating that the temperature will accelerate the racemization of target alkaloids. It is suggested that when tropane alkaloids are extracted, a reasonable temperature range should be selected; continuous ultrasonic extraction will obviously produce heating effects, and too high temperature can lead to a greater degree of racemization of such alkaloids. It is necessary to control temperature during extraction. On the other hand, the optical rotation of anisodamine + ionic liquid aqueous solution under ultrasonic irradiation is higher than that in water bath, which should be ascribed to the fact that the alkaloid molecules can be better dispersed in [C_3_Tr][PF_6_] + H_2_O system and combine with the ionic 1iquid.

### 2.8. Evaluation on Potential Toxicity of [C_3_Tr][PF_6_]

With the advantages of low vapor pressure and easy recovery, ionic liquids are called “green solvents” because they do not introduce or form volatile organics (VOCs) during their use. However, some researchers hold the opinion that ionic liquids are not entirely “green solvents” as some ILs are not environmentally-friendly and even toxic. Therefore, the potential risks should be evaluated before any large-scale application of ionic liquids. In current studies on toxicity evaluation of ILs (especially those with zebrafish as model animal) [18,19] the results usually show that the toxicity of ionic liquids with the same cationic structure varies little, and the effect of anions is obvious only when they are strongly acidic [20]. With the increase of alkyl chain length on cations, the toxicity of ILs with the same anionic structure will become higher. The increase of alkyl chain leads to an increase of lipophilicity and the permeability of cell membrane, which results in stronger biological toxicity. As for the IL selected in this study, [C_3_Tr][PF_6_] not only has no longer alkyl chain (≥C_6_) on its cation, but also has not strongly acidic anions just like [HSO_4_]^−^, [H_2_PO_4_]^−^ [Br]^−^ or [p-TSA]^−^, it is expected to exhibit low toxicity. In order to prove this hypothesis, 10 healthy zebrafish (length: 30 ± 5 mm, weight: 0.3 ± 0.1 g) were selected for an acute toxicity test and the values of median lethal concentration (LC_50_) were measured, using the same operation procedures as those in our previous report [21]. On the basis of the corresponding standards in the China test method classification for acute fish toxicity of dangerous chemicals (GB/T 21281-2007) and International Organization for Standardization (ISO) [22,23], if the LC_50_ value is more than 100 mg/L, so the toxicity of the sample solution under test is considered to be low and there is no fatal effect. As a result, the LC_50_ values (96 h) of [C_3_Tr][PF_6_] is determined as 296.3 ± 2.5 mg/L; as the most common immidazolium ILs, the LC_50_ values (96 h) of [Hmim][HSO_4_], [PSmim][HSO_4_], [C_4_mim][H_2_PO_4_] and [C_3_mim][PF_6_] are 23.1 ± 1.2 mg/L, 24.2 ± 0.2 mg/L, 38.7 ± 1.1 mg/L and 287.1 ± 1.9 mg/L, respectively. Through the comparison, it can be found the toxicity of [C_3_Tr][PF_6_] is not only lower than that of those acidic ILs used in the extraction of alkaloids before [7], but also lower than that of the imidazolium IL with the same length of substituted alkyl chain and anion. Besides that, the diverse combination of various cations and anions will result in the difference of bioaccumulation and degradation among these ILs; the degradation rate is generally accelerated by the presence of alkyl or ester groups on cations. The type and volume of anions have little effect on the degradation of ILs, and the influential trend is not obvious. This may be related to the electrostatic interaction between anions and cations. Moreover, tropine-type ILs also have good biocompatibility, which will not affect the secondary structure of protein in their use [24]. On the other hand, highly efficient recovery is another solution to eliminate potential impact of ILs on environment; meanwhile it can further decrease the cost and expense for repeated extraction.

## 3. Materials and Methods

### 3.1. Reagents and Instruments

In this study all involved chemicals were at least of analytical grade and provided by Kelong Chemical Reagents Factory (Chengdu, China). All the tropane alkaloids standards (purity ≥ 98.0%) used for quantitation were obtained from National Institutes for Food and Drug Control (NIFDC, Beijing, China) or Research Chemicals Inc. (Toronto, ON, Canada). Chromatographic methanol used in high performance liquid chromatography (HPLC) was purchased from Chengdu Chemical Reagents Factory (Chengdu, China), which was filtered with 0.45 μm microporous membrane (Jinteng Inc., Tianjin, China) before analysis. Experimental deionized water was produced by ultra-pure water purification system with 0.4 mm filter (Millipore Co., Ltd., Bedford, MA, USA). Ionic liquids (purity ≥ 97.0%) were prepared according to our developed method [12] and checked by HPLC. Herbal materials of *Radix physochlainae* originating in Shaanxi Province were obtained from a local pharmacy and all samples were stored in closed desiccators until use.

Extraction experiments were carried out using a KQ-2200DA ultrasonic extractor (Shumei Co. Ltd., Jiangsu, China). An EC2006 HPLC system (Elite, Dalian, China) coupled with a UV1201 UV-Vis detector together with 2800 type UV-Vis spectrometer (Hengping Instrument Co. Ltd., Shanghai, China) was employed in quantitation. A Nicolet 6700 Fourier transform infrared spectrometer (FT-IR, Thermo Scientific Co. Ltd., Madison, WI, USA), and JSM-7001F scanning electron microscopy (SEM, JEOL, Tokyo, Japan) were used for the characterization of herbal materials before and after extraction, NIR spectrometer (NIRQUEST512), deuterium light source (DH-2000-BAL) and fiber optic probes (T300-UVVIS) were provided by Ocean Optics (Dunedin, FL, USA). Autopol IV polarimeter (Rudolph Research Analytical, Hackettstown, NJ, USA) was used to explore racemization phenomenon and measure optical rotation values.

### 3.2. Ultrasonic Extraction Process

Tropane alkaloids in *Radix physochlainae* were extracted by ionic liquid aqueous solution according to the following procedures: a certain volume of ionic liquid was placed in 50 mL conical bottle, and 10 mL deionized water was transferred into the conical bottle by the pipette and dissolved the IL completely with ultrasonic wave. 1 g of ground and sieved (60 mesh) powders of *Radix physochlainae* were weighed and mixed thoroughly with the IL aqueous solution. Finally, the conical bottle was sealed and placed in the ultrasonic extractor for 40 min at 100 W. After the extraction was completed, water in the solution was removed under vacuum, and then the residue was dissolved in methanol and its volume adjusted to 10 mL. The sample solution passed through 0.45 microporous filter membrane and 10 μL filtrate was sampled and injected into HPLC for lquid chromatographic analysis based on the reported method [25] for five main alkaloids including anisodamine, atropine, scopolamine, aposcopolamine and scopoline. A Welch C_18_ chromatographic column (4.6 × 250 mm, 5 μm) was used at a column temperature of 25 °C; the mobile phase was composed of 20 mmol/L ammonium acetate solution (A) and methanol (B) and the flow rate was 1.0 mL/min. Detection wavelength was set at 210 nm and injection volume was 5 μL, and the gradient procedure was operated as: 0~10 min, A:B = 6:4 (V/V); 10~30 min, A:B = 3:7 (V/V); after 30 min, A:B = 2:8 (V/V). As a result, the working standard curves of anisodamine, atropine, scopolamine, aposcopolamine and scopoline were determined as y_1_ = 12261.40x_1_ − 368.19 (R^2^ = 0.9998), y_2_ = 3560.60x_2_ − 619.55 (R^2^ = 0.9997), y_3_ = 1628.80x_3_ − 135.65 (R^2^ = 0.9999), y_4_ = 2188.80x_4_ − 266.52 (R^2^ = 0.9997) and y_5_ = 110.59x_5_ − 1.84 (R^2^ = 0.9998), successively, where x was their amount (μg) and y was the UV absorbance value of related peak area, respectively. The methodology validation proved above analytical conditions exhibited good linearity, recovery and precision. The straight lines with regression coefficients were all above 0.9995; the recovery of the alkaloids was between 95.6% and 103.8%, respectively; the intra-day and inter-day RSD of peak area for precision test were 2.18~2.35% and 2.79~3.02%, respectively. Finally, the total amount of these constituents (m_1_, mg) in extraction solution was used to calculate the extraction efficiency (%), which could be determined through the following equation:

Extraction efficiency (E, %) = (m_1_/m_0_) × 100

where m_0_ (mg) is the total amount of these alkaloids amount in herbal powders determined under the extraction conditions in the Chinese Pharmacopoeia (Ch.p., 2015 edition) for quantitation of target alkaloids (1:50 g/mL methanol, 300 W ultrasound, 1 h). All of the data for calculation were the average values obtained from three repeated measurement.

### 3.3. Characterization of Herbal Materials with Various Methods

#### 3.3.1. Scanning Electron Microscopy (SEM) of the Raw Material and Residue after Extraction

The former and latter resulting from ultrasonic extraction in three solvents were crushed and ground, then dried under vacuum for 24 h. The surface morphology of various samples before and after three solvents extraction was observed under SEM with operating voltage of 20 kV and the resolution of 1 nm. The entire sample surface was sprayed with gold for ~100 s as pretreatment.

#### 3.3.2. Infrared Characterization (IR)

Dried powder of raw material and residue after ultrasonic extraction by three solvents (2 mg) were weighed respectively, and then they were mixed with dry KBr powder (150 mg) and pressed into thin tablets, which were scanned by the FT-IR spectrometer from 400 to 4000 cm^−1^. The signal was scanned and accumulated for 32 times in each experiment.

#### 3.3.3. Near Infrared (NIR) Detection for Ultrasonic Extracts

The process analysis experiments were carried out using an NIRQUEST 512 type spectrometer (spectral resolution: 3 nm, detection signal-to-noise ratio: 4000:1, Ocean Optics), and 0.03 mol/L ionic liquid aqueous solution and its ultrasonic extract for 15 and 30 min were detected. The wavelength was in the range of 800~1700 nm, and the optical length of the immersion probe was 1 cm.

### 3.4. Optical Rotation under Ultrasound Conditions

An Autopol IV automatic polarimeter (Rudolph) was used in this investigation. Ionic liquid aqueous solution (0.03 mol/L), standard aqueous solution of scopolamine (1 g/L), aqueous solution of scopolamine-ionic liquid mixture (1 g/L) and ammonia aqueous solution of scopolamine (1 g/L, pH = 9) [9] were prepared and detected, respectively. The optical rotation of the three samples containing scopolamine was measured after ultrasonic irradiation (90 W) for 30 min (these conditions were same as those of the ultrasonic extraction). The detection wavelength was 589 nm, and the testing temperature were 25 °C, 35 °C and 50 °C.

### 3.5. Recovery of IL and Target Products

At the end of ultrasound-assisted extraction, the extract solution was firstly filtered in order to remove the suspended herbal powder. After the removal of water under vacuum, back extraction with a common solvent was selected to separate alkaloids from the ionic liquid and realize their respective recovery, which was frequently employed for the simple and effective operation. In detail, *n*-butyl alcohol was thoroughly mixed with the solid extract with solid–liquid ratio of 1:4 (g/mL) under stirring, then the *n*-butyl alcohol solution containing alkaloids was obtained by filtration and then further concentrated under vacuum. The dissolved residue was recrystallized in methanol to obtain pure [C_3_Tr][PF_6_] for the next use. In previous studies, chloroform was usually to resolve this kind of lipid-soluble alkaloids; however, it was replaced with *n*-butyl alcohol due to the lower toxicity of the latter.

## 4. Conclusions

According to our research, N-propyltropine hexafluorophosphate ([C_3_Tr][PF_6_]) was found most effective in the ultrasound extraction of tropane alkaloids from *Radix physochlainae*, which was superior to traditional solvents and non-tropine ILs. The effect of ILs on the optical activity of target alkaloids was studied and revealed the absence of optically active substances in an aqueous solution of IL and therefore, the use of ionic liquid in the presence of anisodamine does not racemize the latter. It was also proven that the IL was safe according to the toxicity investigation. Moreover, scanning electron microscopy, medium and near Infrared detection were used to analyze the potential change of the extract system composed of solid and liquid phases. In comparison to conventional heating mode, ultrasonic extraction shortens the extraction time, improves the extraction efficiency and minimize the IL consumption as well as subsequent back-extraction could be used to remove coexisting non-alkaloid constituents.

## Figures and Tables

**Table 1:** Structures, chemical names and acid-base properties of the eight studied ILs.

Entry	IL Structure	IL Abbr.	Chemical Name	Acidity/Alkalinity *
1	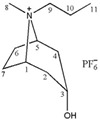	[C_3_Tr][PF_6_]	N-Propyltropine hexafluorophosphate	Neutral
2	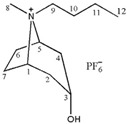	[C_4_Tr][PF_6_]	N-Butyltropine hexafluorophosphate	Neutral
3	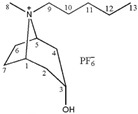	[C_5_Tr][PF_6_]	N-Amyltropine hexafluorophosphate	Neutral
4	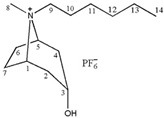	[C_6_Tr][PF_6_]	N-Hexyltropine hexafluorophosphate	Neutral
5	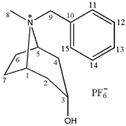	[Bntr][PF_6_]	N-Benzyltropine hexafluorophosphate	Neutral
6	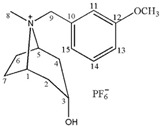	[m-MBntr][PF_6_]	N-(*m*-Methoxybenzyl)-tropine hexafluorophosphate	Neutral
7	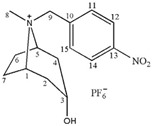	[p-NBntr][PF_6_]	N-(*p*-Nitrobenzyl)-tropine hexafluorophosphate	Neutral
8	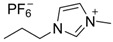	[C_3_mim][PF_6_]	1-Propyl-3-methyl-imidazolium hexafluorophosphate	Neutral

* Acid-base properties of above ionic liquids were judged based on their 0.03 mol/L aqueous solutions at room temperature using pH test paper.

**Table 2:** The molar conductivities of seven tropine-type ionic liquids in aqueous solution.

T	10^4^ C (mol·L^−1^)	Λ (S·cm^2^·mol^−1^) *			
		[C_3_tr][PF_6_]	[C_4_tr][PF_6_]	[C_5_tr][PF_6_]	[C_6_tr][PF_6_]
5 °C	10.0000	59.08	54.09	49.88	47.87
	9.0909	59.27	54.27	50.02	48.09
	8.3333	59.44	54.42	50.14	48.28
	7.6923	59.58	54.55	50.24	48.43
	7.1429	59.70	54.66	50.33	48.57
	6.6667	59.80	54.75	50.41	48.69
	6.0000	59.95	54.89	50.52	48.86
	5.4545	60.07	55.00	50.61	49.00
	5.0000	60.18	55.10	50.69	49.12
	4.6154	60.26	55.18	50.75	49.22
	4.0000	60.41	55.31	50.85	49.39
	3.5294	60.52	55.41	50.93	49.51
	3.1579	60.60	55.49	51.00	49.62
	2.6087	60.73	55.60	51.09	49.77
	2.2222	60.82	55.69	51.16	49.87
	1.8182	60.92	55.78	51.23	49.99
	1.3953	61.02	55.87	51.30	50.11
45 °C	10.0000	121.41	116.10	113.37	111.25
	9.0909	122.10	116.93	114.06	111.85
	8.3333	122.68	117.48	114.64	112.36
	7.6923	123.18	118.30	115.14	112.80
	7.1429	123.61	118.72	115.58	113.18
	6.6667	123.99	119.10	115.97	113.52
	6.0000	124.53	119.67	116.51	113.99
	5.4545	124.98	120.08	116.97	114.39
	5.0000	125.36	120.60	117.35	114.72
	4.6154	125.69	120.90	117.68	115.01
	4.0000	126.21	121.25	118.22	115.47
	3.5294	126.62	121.55	118.63	115.83
	3.1579	126.95	121.92	118.97	116.11
	2.6087	127.44	122.28	119.46	116.54
	2.2222	127.79	122.85	119.82	116.84
	1.8182	128.16	123.20	120.20	117.17
	1.3953	128.55	123.98	120.60	117.51
65 °C	10.0000	154.52	138.90	135.90	133.49
	9.0909	155.67	140.36	136.86	134.40
	8.3333	156.66	141.12	137.69	135.18
	7.6923	157.52	141.57	138.40	135.86
	7.1429	158.27	142.10	139.03	136.45
	6.6667	158.93	142.65	139.58	136.97
	6.0000	159.87	143.33	140.36	137.71
	5.4545	160.66	143.92	141.02	138.33
	5.0000	161.33	144.60	141.57	138.85
	4.6154	161.90	145.17	142.05	139.30
	4.0000	162.84	145.50	142.82	140.03
	3.5294	163.57	146.77	143.43	140.59
	3.1579	164.15	146.93	143.91	141.05
	2.6087	165.03	148.73	144.64	141.73
	2.2222	165.66	149.85	145.16	142.22
	1.8182	166.34	152.35	145.71	142.74
	1.3953	167.05	154.08	146.30	143.29
85 °C	10.0000	231.05	228.37	215.57	199.14
	9.0909	233.38	230.04	217.65	200.88
	8.3333	235.39	231.48	219.44	202.36
	7.6923	237.13	232.72	221.00	203.66
	7.1429	238.68	233.80	222.37	204.79
	6.6667	240.05	234.76	223.58	205.79
	6.0000	242.02	236.12	225.33	207.23
	5.4545	243.68	237.26	226.79	208.43
	5.0000	245.09	238.23	228.05	209.46
	4.6154	246.31	239.06	229.13	210.34
	4.0000	248.32	240.41	230.90	211.78
	3.5294	249.90	241.46	232.29	212.91
	3.1579	251.18	242.31	233.41	213.82
	2.6087	253.11	243.58	235.11	215.19
	2.2222	254.50	244.49	236.33	216.18
	1.8182	255.99	245.46	237.64	217.23
	1.3953	257.59	246.48	239.04	218.35

* Basic error: ±1.0%, match error: ±1.5%.

**Table 3:** Limiting molar conductivities and association constants of [C_3_Tr][PF_6_]-alkaloid at different concentrations and temperatures.

Concentration g/mL	Λ^∞^ S·cm^2^·mol^−1^	K_A_ L·mol^−1^	Λ^∞^ S·cm^2^·mol^−1^	K_A_ L·mol^−1^	Λ^∞^ S·cm^2^·mol^−1^	K_A_ L·mol^−1^
	303.15K		313.15K		323.15K	
0.000	79.365	18.896	116.279	40.562	156.250	48.828
0.225	87.719	23.084	117.647	41.522	169.492	57.455
0.451	90.090	24.349	135.135	54.785	175.439	61.557
0.676	96.899	28.278	120.481	43.548	166.670	55.556
0.901	91.743	25.250	114.942	39.635	161.290	52.029
1.126	90.909	24.793	116.279	40.562	158.730	50.391
	333.15 K		343.15 K			
0.000	192.308	73.965	208.333	86.806		
0.225	200.000	80.000	227.272	103.306		
0.451	212.766	90.539	212.766	90.539		
0.676	196.078	76.894	212.766	86.806		
0.901	192.308	76.894	217.391	94.518		
1.126	163.934	53.745	217.391	94.518		

**Table 4:** Effect of different extractants on content of scopoletin.

pH	Content of Scopoletin in the Extract (mg/g)		
	80% EtOH	85% EtOH	0.03 mol/L [C_3_tr][PF_6_]
3	1.20 ± 0.02	1.21 ± 0.02	1.09 ± 0.02
5	1.08 ± 0.02	1.12 ± 0.02	1.08 ± 0.02
7	1.06 ± 0.02	1.09 ± 0.02	1.05 ± 0.02

**Table 5:** Values of optical rotation of four samples at different temperatures after mixing for 30 min under 90 W ultrasonic waves.

Test Samples	Optical Rotation at Various Temperatures (Mean Value of Three Parallel Experiments)		
	25 °C	35 °C	50 °C
[C_3_Tr][PF_6_] + H_2_O	0.000	0.000	0.000
Anisodamine + H_2_O	−0.029	−0.023	−0.020
Anisodamine+[C_3_Tr][PF_6_] + H_2_O	−0.029	−0.023	−0.019
Anisodamine + NH_3_·H_2_O	−0.016	−0.014	−0.008
